# Supplementary material for: Telehealth Use in Geriatrics Care during the COVID-19 Pandemic—A Scoping Review and Evidence Synthesis
Source: Int J Environ Res Public Health. 2021 Feb 11;18(4):1755. doi: 10.3390/ijerph18041755 (PMC7918552; doi:10.3390/ijerph18041755)
Supplement: Supplementary file 1 [file ijerph-18-01755-s001.zip › Supplementary files_updated/File S2 - Telehealth and geriatric care.docx]

**Annex 2: Search strategy**

**PubMed**

| **Search No** | **Query** | **Results** | **Date** |
| --- | --- | --- | --- |
| 1 | #2 AND #3 | 323 | 20-Aug-20 |
| 2 | (((((("covid 19"[All Fields] OR "covid 2019"[All Fields]) OR "severe acute respiratory syndrome coronavirus 2"[Supplementary Concept]) OR "severe acute respiratory syndrome coronavirus 2"[All Fields]) OR "2019 ncov"[All Fields]) OR "sars cov 2"[All Fields]) OR "2019ncov"[All Fields]) OR (("wuhan"[All Fields] AND ("coronavirus"[MeSH Terms] OR "coronavirus"[All Fields])) AND (2019/12/1:2019/12/31[Date - Publication] OR 2020/1/1:2020/12/31[Date - Publication])) | 42,010 | 20-Aug-20 |
| 3 | #4 AND #8 | 113,541 | 20-Aug-20 |
| 4 | #5 OR #6 OR #7 | 712,058 | 20-Aug-20 |
| 5 | "tele*"[All Fields] OR "digital*"[All Fields] OR "remote*"[All Fields] OR "video*"[All Fields] OR "Ehealth"[All Fields] OR "e-health"[All Fields] OR "e-health"[All Fields] OR "electronic health"[All Fields] OR "virtual"[All Fields] OR "virtuality"[All Fields] OR "virtualization"[All Fields] OR "virtualized"[All Fields] OR "virtualizing"[All Fields] OR "virtuals"[All Fields] | 712,058 | 20-Aug-20 |
| 6 | mobile health [MeSH Terms] | 29,271 | 20-Aug-20 |
| 7 | (telemedicine [MeSH Terms]) OR (telehealth [MeSH Terms]) | 29,271 | 20-Aug-20 |
| 8 | #9 OR #10 | 3,514,343 | 20-Aug-20 |
| 9 | senior or senile or gerontol* | 170,794 | 20-Aug-20 |
| 10 | ("geriatrics"[MH] OR "geriatric*"[TW] OR "aged"[MH] OR "aged, 80 and over"[MH] OR "elderly"[TW] OR "older"[TW] OR "Aged: 65+ years"[TW]) | 3,427,289 | 20-Aug-20 |

**EMBASE**

| **Search No** | **Query** | **Results** | **Date** |
| --- | --- | --- | --- |
| 1 | exp telehealth/ | 47,276 | 20-Aug-20 |
| 2 | exp telemedicine/ | 41,217 | 20-Aug-20 |
| 3 | (tele* or digital* or remote or video* or Ehealth or e-health or "electronic health" or virtual).mp. [mp=title, abstract, heading word, drug trade name, original title, device manufacturer, drug manufacturer, device trade name, keyword, floating subheading word, candidate term word] | 818,825 | 20-Aug-20 |
| 4 | 1 or 2 or 3 | 818,825 | 20-Aug-20 |
| 5 | limit 4 to exclude medline journals | 72,026 | 20-Aug-20 |
| 6 | geriatrics.mp. or exp geriatrics/ | 44,549 | 20-Aug-20 |
| 7 | elderly.mp. | 546,236 | 20-Aug-20 |
| 8 | exp aged/ or aged.mp. | 4,287,513 | 20-Aug-20 |
| 9 | older.mp. | 593,211 | 20-Aug-20 |
| 10 | senior.mp. or exp elderly care/ or exp nebivolol/ | 121,662 | 20-Aug-20 |
| 11 | gerontology.mp. or exp gerontology/ | 6,631 | 20-Aug-20 |
| 12 | gerontol*.mp. | 13,615 | 20-Aug-20 |
| 13 | 6 or 7 or 8 or 9 or 10 or 11 or 12 | 4,658,235 | 20-Aug-20 |
| 14 | 5 and 13 | 13,973 | 20-Aug-20 |
| 15 | limit 14 to covid-19 | 56 | 20-Aug-20 |

**WHO**

| **Search No** | **Query** | **Results** | **Date** |
| --- | --- | --- | --- |
| 1 | (tw:(geriatr*)) OR (tw:(gerontol*)) OR (tw:(elder*)) OR (tw:(old*)) OR (tw:(aged)) OR (tw:(senior*)) OR (tw:(senile)) | 5,715 | 20-Aug-20 |
| 2 | (tw:(tele*)) OR (tw:(digital*)) OR (tw:(remote*)) OR (tw:(video*)) OR (tw:(ehealth)) OR (tw:(e-health)) OR (tw:(virtual*)) | 4,177 | 20-Aug-20 |
| 3 | ((tw:(tele*)) OR (tw:(digital*)) OR (tw:(remote*)) OR (tw:(video*)) OR (tw:(ehealth)) OR (tw:(e-health)) OR (tw:(virtual*))) AND ((tw:(geriatr*)) OR (tw:(gerontol*)) OR (tw:(elder*)) OR (tw:(old*)) OR (tw:(aged)) OR (tw:(senior*)) OR (tw:(senile))) | 427 | 20-Aug-20 |
