# Supplementary material for: Telehealth Use in Geriatrics Care during the COVID-19 Pandemic—A Scoping Review and Evidence Synthesis
Source: Int J Environ Res Public Health. 2021 Feb 11;18(4):1755. doi: 10.3390/ijerph18041755 (PMC7918552; doi:10.3390/ijerph18041755)
Supplement: Supplementary file 1 [file ijerph-18-01755-s001.zip › Supplementary files_updated/File S3 - Telehealth and geriatric care.docx]

**ANNEX 4: World Bank Classification of Countries**

| **Income group** | **Country** | **Number** | **Frequency** |
| --- | --- | --- | --- |
| Low-income countries | N/A | N/A | N/A |
| Lower-middle income countries | India | 2 | 2.5 |
| Upper-middle income countries | China | 2* | 2.5 |
|  | Mexico | 1 | 1.3 |
| High income countries | USA | 34 | 43 |
|  | UK | 5 | 6.3 |
|  | Italy | 5 | 6.3 |
|  | Canada | 7 | 8.9 |
|  | Australia | 2 | 2.5 |
|  | France | 3 | 3.8 |
|  | Spain | 5 | 6.3 |
|  | Switzerland | 2 | 2.5 |
|  | Germany | 1 | 1.3 |
|  | Ireland | 1 | 1.3 |
|  | Israel | 1 | 1.3 |
|  | Japan | 1 | 1.3 |
|  | South Korea | 1 | 1.3 |
|  | Netherlands | 1 | 1.3 |
|  | New Zealand | 1 | 1.3 |
|  | Poland | 1 | 1.3 |
|  | Chile | 1 | 1.3 |
|  | Multiple Countries | 2 | 2.5 |

*Including Hong Kong, Special Administrative Region of the People’s Republic of China

**World Health Organization Regional Classification of Countries**

| **Region** | **Country** | **Number** | **Frequency** |
| --- | --- | --- | --- |
| Africa | N/A | N/A | N/A |
| Americas | USA | 34 | 43 |
|  | Canada | 7 | 8.9 |
|  | Mexico | 1 | 1.3 |
|  | Chile | 1 | 1.3 |
| Europe | UK | 5 | 6.3 |
|  | Italy | 5 | 6.3 |
|  | France | 3 | 3.8 |
|  | Spain | 5 | 6.3 |
|  | Switzerland | 2 | 2.5 |
|  | Germany | 1 | 1.3 |
|  | Ireland | 1 | 1.3 |
|  | Netherlands | 1 | 1.3 |
|  | Poland | 1 | 1.3 |
| Eastern Mediterranean | Israel | 1 | 1.3 |
| South East Asia | India | 2 | 2.5 |
| Western Pacific | Australia | 2 | 2.5 |
|  | China | 2* | 2.5 |
|  | Japan | 1 | 1.3 |
|  | New Zealand | 1 | 1.3 |
|  | South Korea | 1 | 1.3 |
|  | Multiple Countries | 2 | 2.5 |

*Including Hong Kong, Special Administrative Region of the People’s Republic of China
