# Supplementary material for: Telehealth Use in Geriatrics Care during the COVID-19 Pandemic—A Scoping Review and Evidence Synthesis
Source: Int J Environ Res Public Health. 2021 Feb 11;18(4):1755. doi: 10.3390/ijerph18041755 (PMC7918552; doi:10.3390/ijerph18041755)
Supplement: Supplementary file 1 [file ijerph-18-01755-s001.zip › Supplementary files_updated/File S4 - Telehealth and geriatric care.docx]

**ANNEX 5: Terminologies used**

| **Number** | **Terminology** | **Number** | **Terminology** |
| --- | --- | --- | --- |
| 1 | Asynchronous video exercise | 26 | Tele mentoring |
| 2 | Digital divide | 27 | Tele neurology |
| 3 | Digital image prescriptions | 28 | Tele‐expertise |
| 4 | Digital learning | 29 | Teleconsultation |
| 5 | Digital tablets | 30 | Tele dentistry |
| 6 | Digital technology | 31 | Telehealth |
| 7 | Digital services | 32 | Telematic Triage |
| 8 | Direct-to-patient telemedicine | 33 | Telematic visits |
| 9 | E-health services | 34 | Telephone clinics |
| 10 | Electronic prescribing (ePrescribing) | 35 | Telephone companion |
| 11 | Evaluation SOcio-GERiatrique” (ESOGER) | 36 | Telephone outreach |
| 12 | Gerontechnology | 37 | Telerehabilitation |
| 13 | Home health care | 38 | Video monitoring |
| 14 | Mobile technology | 39 | Video-telehealth |
| 15 | Non-face-to-face encounters | 40 | Videoconferencing |
| 16 | Online technology | 41 | Virtual care |
| 17 | Remote care | 42 | Virtual communication |
| 18 | Remote consultations | 43 | Virtual dermatologic care |
| 19 | Remote counseling | 44 | Virtual funeral services |
| 20 | Remote interventions | 45 | Virtual psychiatric care |
| 21 | Remote memorial services | 46 | Virtual psychotherapy (e‐therapies) |
| 22 | Remote monitoring | 47 | Virtual reality |
| 23 | Remote offerings | 48 | Virtual visits |
| 24 | Tele dermatology | 49 | Virtual psychotherapy (e‐therapies) |
| 25 | Tele medicine |  |  |
