# Supplementary material for: Telehealth Use in Geriatrics Care during the COVID-19 Pandemic—A Scoping Review and Evidence Synthesis
Source: Int J Environ Res Public Health. 2021 Feb 11;18(4):1755. doi: 10.3390/ijerph18041755 (PMC7918552; doi:10.3390/ijerph18041755)
Supplement: Supplementary file 1 [file ijerph-18-01755-s001.zip › Supplementary files_updated/File S5 - Telehealth and geriatric care.docx]

**ANNEX 3: List of included studies**

**Strengths**

1. Battisti NML, Mislang AR, Cooper L, Donovan A, Audisio RA, Cheung K-L, et al. Adapting care for older cancer patients during the COVID-19 pandemic: Recommendations from the International Society of Geriatric Oncology (SIOG) COVID-19 Working Group. Journal of geriatric oncology. 2020
2. Chatterjee P, Yatnatti SK. Intergenerational Digital Engagement: A Way to Prevent Social Isolation During the COVID-19 Crisis. J Am Geriatr Soc.68(7):1394-5
3. Hau YS, Kim JK, Hur J, Chang MC. How about actively using telemedicine during the COVID-19 pandemic? J Med Syst.44(6):108
4. Longpré-Poirier C, Desbeaumes Jodoin V, Miron J-P, Lespérance P. &quot;Remote Monitoring of Intranasal Ketamine Self-Administration as Maintenance Therapy in Treatment-Resistant Depression (TRD): A Novel Strategy for Vulnerable and At-Risk Populations to COVID-19?&quot. Am J Geriatr Psychiatry. 2020;28(8):892-3
5. Ishikawa RZ. I may never see the ocean again: Loss and grief among older adults during the COVID-19 pandemic. Psychol Trauma.12:S85-s6
6. Cheung G, Peri K. Challenges to dementia care during COVID-19: Innovations in remote delivery of group Cognitive Stimulation Therapy. Taylor & Francis; 2020.
7. Cuffaro L, Di Lorenzo F, Bonavita S, Tedeschi G, Leocani L, Lavorgna L. Dementia care and COVID-19 pandemic: a necessary digital revolution. Neurol Sci. 2020;41(8):1977-9
8. Canevelli M, Bruno G, Cesari M. Providing Simultaneous COVID-19-sensitive and Dementia-Sensitive Care as We Transition from Crisis Care to Ongoing Care. J Am Med Dir Assoc. 2020;21(7):968-9
9. Carr D, Boerner K, Moorman S. Bereavement in the Time of Coronavirus: Unprecedented Challenges Demand Novel Interventions. J Aging Soc Policy.32(4):425-31
10. Chen AT, Ge S, Cho S, Teng AK, Chu F, Demiris G, et al. Reactions to COVID-19, Information and Technology Use, and Social Connectedness among Older Adults with Pre-Frailty and Frailty. Geriatric Nursing. 2020
11. Escura Sancho S, Ros Cerro C, Angles-Acedo S, Bataller Sanchez E, Espuna-Pons M. How did COVID-19 pandemic change the way we attend the patients in an urogynaecological unit. Clinica e Investigacion en Ginecologia y Obstetricia. 2020
12. Cormi C, Chrusciel J, Laplanche D, Dramé M, Sanchez S. Telemedicine in nursing homes during the COVID-19 outbreak: A star is born (again). Geriatr Gerontol Int.20(6):646-7
13. Fatyga E, Dziegielewska-Gesiak S, Wierzgon A, er, Stoltny D, Muc-Wierzgon M. The coronavirus disease 2019 pandemic: telemedicine in elderly patients with type 2 diabetes. Pol Arch Intern Med. 2020;130(5):452-4
14. McLellan LJ, Morelli M, Simeone E, Khazova M, Ibbotson SH, Eadie E. SmartPDT®: smartphone enabled real-time dosimetry via satellite observation for daylight photodynamic therapy. Photodiagnosis Photodyn Ther.31:101914

**Weakness**

1. DiGiovanni G, Mousaw K, Lloyd T, Dukelow N, Fitzgerald B, D'Aurizio H, et al. Development of a telehealth geriatric assessment model in response to the COVID-19 pandemic. J Geriatr Oncol.11(5):761-3
2. Fisk M, Livingstone A, Pit SW. Telehealth in the Context of COVID-19: Changing Perspectives in Australia, the United Kingdom, and the United States. J Med Internet Res.22(6):e19264
3. Siette J, Wuthrich V, Low L-F. Social Preparedness in Response to Spatial Distancing Measures for Aged Care During COVID-19. J Am Med Dir Assoc. 2020;21(7):985-6
4. Kasle DA, Torabi SJ, Savoca EL, Judson BL, Manes RP. Outpatient Otolaryngology in the Era of COVID-19: A Data-Driven Analysis of Practice Patterns. Otolaryngol Head Neck Surg.163(1):138-44
5. Benaque A, Gurruchaga MJ, Abdelnour C, Hernández I, Cañabate P, Alegret M, et al. Dementia Care in Times of COVID-19: Experience at Fundació ACE in Barcelona, Spain. J Alzheimers Dis. 2020;76(1):33-40
6. Marra DE, Hamlet KM, Bauer RM, Bowers D. Validity of teleneuropsychology for older adults in response to COVID-19: A systematic and critical review. Clin Neuropsychol.1-42
7. Dewar S, Lee PG, Suh TT, Min L. Uptake of Virtual Visits in A Geriatric Primary Care Clinic During the COVID-19 Pandemic. J Am Geriatr Soc.68(7):1392-4
8. Lipner SR, Ouchida K. Novel Solutions for Dermatologic Care of Geriatric Patients and the Role of the Dermatology House Call. J Am Acad Dermatol
9. Patel S, Gannon A, Dolan C, McCarthy G. Telehealth in Psychiatry of Old Age: Ordinary Care in Extraordinary Times in Rural North-West Ireland. Am J Geriatr Psychiatry.28(9):1009-11
10. Tousi B. Dementia Care in the Time of COVID-19 Pandemic. J Alzheimers Dis. 2020;76(2):475-9
11. Korczyn AD. Dementia in the COVID-19 Period. J Alzheimers Dis. 2020;75(4):1071-2
12. Phillips NA, Chertkow H, Pichora-Fuller MK, Wittich W. Special Issues on Using the Montreal Cognitive Assessment for telemedicine Assessment During COVID-19. J Am Geriatr Soc.68(5):942-4
13. Sano M, Lapid M, Ikeda M, Mateos R, Wang H, Reichman WE. Psychogeriatrics in a World with COVID-19. International Psychogeriatrics. 2020:1-9
14. Seifert A. The Digital Exclusion of Older Adults during the COVID-19 Pandemic. J Gerontol Soc Work.1-3
15. Simpson CL, Kovarik CL. Effectively Engaging Geriatric Patients via Teledermatology. J Am Acad Dermatol
16. Luciani LG, Mattevi D, Cai T, Giusti G, Proietti S, Malossini G. Teleurology in the Time of Covid-19 Pandemic: Here to Stay? Urology. 2020;140:4-6

**Opportunities**

1. Banskota S, Healy M, Goldberg EM. 15 Smartphone Apps for Older Adults to Use While in Isolation During the COVID-19 Pandemic. West J Emerg Med. 2020;21(3):514-25
2. León S, Giacaman RA. COVID-19 and Inequities in Oral Health Care for Older People: An Opportunity for Emerging Paradigms. JDR Clin Trans Res.2380084420934742
3. Edelman LS, McConnell ES, Kennerly SM, Alderden J, Horn SD, Yap TL. Mitigating the Effects of a Pandemic: Facilitating Improved Nursing Home Care Delivery Through Technology. JMIR Aging.3(1):e20110
4. Chen K. Use of Gerontechnology to Assist Older Adults to Cope with the COVID-19 Pandemic. J Am Med Dir Assoc.21(7):983-4
5. Yu EW, Tsourdi E, Clarke BL, Bauer DC, Drake MT. Osteoporosis Management in the Era of COVID-19. J Bone Miner Res. 2020;35(6):1009-13
6. Morrow-Howell N, Galucia N, Swinford E. Recovering from the COVID-19 Pandemic: A Focus on Older Adults. J Aging Soc Policy. 2020;32(4):526-35
7. Upadhyaya GK, Iyengar K, Jain VK, Vaishya R. Challenges and strategies in management of osteoporosis and fragility fracture care during COVID-19 pandemic. Journal of Orthopaedics. 2020;21:287-90
8. Roy J, Jain R, Golamari R, Vunnam R, Sahu N. COVID-19 in the geriatric population. Int J Geriatr Psychiatry
9. Lai FH-y, Yan EW-h, Chan DT-h. The Protective Impact of Telemedicine on Persons with Dementia and their Caregivers During the COVID-19 Pandemic. The American Journal of Geriatric Psychiatry. 2020
10. Sorinmade OA, Kossoff L, Peisah C. COVID‐19 and Telehealth in Older Adult Psychiatry‐opportunities for now and the Future. International journal of geriatric psychiatry. 2020
11. Goulabch, R, Bocle H, Vignet R, Sotto A, Loubet P. Digital tablets to improve quality of life of COVID-19 older inpatients during lockdown. European Geriatric Medicine. 2020
12. Goodman-Casanova JM, Dura-Perez E, Guzman-Parra J, Cuesta-Vargas A, Mayoral-Cleries F. Telehealth Home Support During COVID-19 Confinement for Community-Dwelling Older Adults With Mild Cognitive Impairment or Mild Dementia: Survey Study. J Med Internet Res.22(5):e19434
13. Naarding P, Oude Voshaar RC, Marijnissen RM. COVID-19: Clinical Challenges in Dutch Geriatric Psychiatry. Am J Geriatr Psychiatry.28(8):839-43
14. Echeverría P, Mas Bergas MA, Puig J, Isnard M, Massot M, Vedia C, et al. COVIDApp as an Innovative Strategy for the Management and Follow-Up of COVID-19 Cases in Long-Term Care Facilities in Catalonia: Implementation Study. JMIR Public Health Surveill. 2020;6(3):e21163-e

**Threats**

1. Archbald-Pannone LR, Harris DA, Albero K, Steele RL, Pannone AF, Mutter JB. COVID-19 Collaborative Model for an Academic Hospital and Long-Term Care Facilities. J Am Med Dir Assoc. 2020;21(7):939-42.
2. Eghtesadi M. Breaking Social Isolation Amidst COVID-19: A Viewpoint on Improving Access to Technology in Long-Term Care Facilities. J Am Geriatr Soc.68(5):949-50.
3. Hartt M. COVID-19: a lonely pandemic. Cities Health. 2020.
4. Hoffman GJ, Webster NJ, Bynum JPW. A Framework for Aging-Friendly Services and Supports in the Age of COVID-19. J Aging Soc Policy.1-10
5. Danilewitz M, Ainsworth NJ, Bahji A, Chan P, Rabheru K. Virtual psychiatric care for older adults in the age of COVID-19: challenges & opportunities. Int J Geriatr Psychiatry.
6. Elbeddini A, Prabaharan T, Almasalkhi S, Tran C, Zhou Y. Barriers to conducting deprescribing in the elderly population amid the COVID-19 pandemic. Res Social Adm Pharm.
7. Frost R, Nimmons D, Davies N. Using Remote Interventions in Promoting the Health of Frail Older Persons Following the COVID-19 Lockdown: Challenges and Solutions. J Am Med Dir Assoc.21(7):992-3
8. Gibson A, Bardach SH, Pope ND. COVID-19 and the Digital Divide: Will Social Workers Help Bridge the Gap? J Gerontol Soc Work.1-3
9. Gould CE, Hantke NC. Promoting Technology and Virtual Visits to Improve Older Adult Mental Health in the Face of COVID-19. Am J Geriatr Psychiatry.28(8):889-90
10. Seifert A, Cotten SR, Xie B. A Double Burden of Exclusion?Digital and Social Exclusion of Older Adults in Times of COVID-19. J Gerontol B Psychol Sci Soc Sci
11. Joy M, McGagh D, Jones N, Liyanage H, Sherlock J, Parimalanathan V, et al. Reorganisation of primary care for older adults during COVID-19: a cross-sectional database study in the UK. Br J Gen Pract.70(697):e540-e7
12. Lam K, Lu AD, Shi Y, Covinsky KE. Assessing Telemedicine Unreadiness Among Older Adults in the United States During the COVID-19 Pandemic. JAMA Intern Med

**General**

1. Nguyen NP, Vinh-Hung V, Baumert B, Zamagni A, Arenas M, Motta M, et al. Older Cancer Patients during the COVID-19 Epidemic: Practice Proposal of the International Geriatric Radiotherapy Group. Cancers (Basel).12(5)
2. Middleton A, Simpson KN, Bettger JP, Bowden MG. COVID-19 Pandemic and Beyond: Considerations and Costs of Telehealth Exercise Programs for Older Adults With Functional Impairments Living at Home-Lessons Learned from a Pilot Case Study. Phys Ther
3. D'Adamo H, Yoshikawa T, Ouslander JG. Coronavirus disease 2019 in geriatrics and long‐term care: the ABCDs of COVID‐19. Journal of the American Geriatrics Society. 2020;68(5):912-7
4. Aung MN, Yuasa M, Koyanagi Y, Aung TNN, Moolphate S, Matsumoto H, et al. Sustainable health promotion for the seniors during COVID-19 outbreak: a lesson from Tokyo. J Infect Dev Ctries.14(4):328-31
5. Hantke NC, Gould C. Examining Older Adult Cognitive Status in the Time of COVID-19. Journal of the American Geriatrics Society. 2020;68(7):1387-9
6. Medina-Walpole A. In COVID-19 response to Congress & Administration, AGS calls for access to medical supplies, telehealth, among other needs. Geriatr Nurs
7. Rorai V, Perry TE. An Innovative Telephone Outreach Program to Seniors in Detroit, a City Facing Dire Consequences of COVID-19. J Gerontol Soc Work.1-4
8. Lester PE, Holahan T, Siskind D, Healy E. Policy Recommendations Regarding Skilled Nursing Facility Management of Coronavirus 19 (COVID-19): Lessons from New York State. J Am Med Dir Assoc.21(7):888-92
9. Fratino L, Procopio G, Di Maio M, Cinieri S, Leo S, Beretta G. Coronavirus: Older Persons With Cancer in Italy in the COVID-19 Pandemic. Frontiers in Oncology. 2020;10:648
10. Bell JS, Reynolds L, Freeman C, Jackson JK. Strategies to promote access to medications during the COVID-19 pandemic. Aust J Gen Pract. 2020;49(8):530-2
11. Beauchet O, Cooper-Brown L, Ivensky V, Launay CP. Telemedicine for housebound older persons during the Covid-19 pandemic. Maturitas. 2020(142):8-10
12. Zubatsky M, Berg-Weger M, Morley J. Using Telehealth Groups to Combat Loneliness in Older Adults Through COVID-19. J Am Geriatr Soc.68(8):1678-9
13. Van Orden KA, Bower E, Lutz J, Silva C, Gallegos AM, Podgorski CA, et al. Strategies to Promote Social Connections Among Older Adults During 'Social Distancing' Restrictions. Am J Geriatr Psychiatry
14. van Dyck LI, Wilkins KM, Ouellet J, Ouellet GM, Conroy ML. Combating Heightened Social Isolation of Nursing Home Elders: The Telephone Outreach in the COVID-19 Outbreak Program. Am J Geriatr Psychiatry.28(9):989-92
15. Office EE, Rodenstein MS, Merchant TS, Pendergrast TR, Lindquist LA. Reducing Social Isolation of Seniors during COVID-19 through Medical Student Telephone Contact. J Am Med Dir Assoc.21(7):948-50
16. Navarrete-Reyes AP, Avila-Funes JA. Staying in a Burning House: Perks and Perils of a Hotline in the Times of COVID-19. J Am Geriatr Soc.68(5):E10-e1
17. Mahoney KJ. Self-Direction of Home and Community-Based Services in the Time of COVID-19. J Gerontol Soc Work.1-4
18. Gao Z, Lee JE, McDonough DJ, Albers C. Virtual Reality Exercise as a Coping Strategy for Health and Wellness Promotion in Older Adults during the COVID-19 Pandemic. J Clin Med.9(6)
19. Gálvez AMP, Romero BB, Trigo SB, Serrano ML. Elderly people, dependecny and vulnerability in the coronavirus oandemic: An emergency for a social and the health integration. Enferm Clin
20. Berrut G, de Decker L, Aquino JP, Ahmine S, Amalberti N, Arlaud C, et al. Geriatric units at the beginning of the 2020 COVID-19 epidemic in France. Geriatr Psychol Neuropsychiatr Vieil.18(2):125-33
21. Gemelli Against COVID-19 Post-Acute Care Study Group. Post-COVID-19 global health strategies: the need for an interdisciplinary approach. Aging clin exp res. 2020
22. Ehni H-J, Wahl H-W. Six Propositions against Ageism in the COVID-19 Pandemic. J Aging Soc Policy. 2020;32(4):515-25
23. Xie B, Charness N, Fingerman K, Kaye J, Kim MT, Khurshid A. When Going Digital Becomes a Necessity: Ensuring Older Adults' Needs for Information, Services, and Social Inclusion During COVID-19. J Aging Soc Policy.1-11.
